# RURAL URBAN DIFFERENCES IN INFORMAL CAREGIVING

**DOI:** 10.1093/geroni/igae098.1821

**Published:** 2024-12-31

**Authors:** Elizabeth Crouch, Monique Brown, Steven Cohen, Jessica Cassidy

**Affiliations:** University of South Carolina, Columbia, South Carolina, United States; University of South Carolina, Columbia, South Carolina, United States; University of Rhode Island, Kingston, Rhode Island, United States; The University of Texas Arlington, Arlington, Texas, United States

## Abstract

With the rapidly rising aging of the U.S. population, an increasing number of stakeholders have raised concerns regarding the health of this group and where the burden of caring for them will fall. The majority of this caregiving will be at the hands of family members and friends who provide informal, unpaid care for their loved ones. In addition to a greater number of older adults who will need care, these adults are also living longer resulting in a greater need for long-term care. The distribution of both formal and informal caregiving in the United States is not uniform; informal caregiving is significantly more prevalent in rural areas than in urban while formal caregiving has the opposite distribution. Greater reliance on informal caregiving in rural areas may be due to a larger proportion of older adults living in rural areas than in urban, reduced access to formal care services in rural areas, or greater feelings of familial obligations amongst rural residents. Prior research has found that the lived experiences of rural caregivers are markedly different from those of their urban counterparts. During this session, we will describe research findings from our team on rural-urban differences in the health and lived experiences of caregivers. We will also discuss interventions that may be tailored to rural communities. This session will help stakeholders and policy makers to better understand the landscape of informal caregiving amongst minoritized rural residents and help program developers design and implement initiatives for rural caregivers.

